# Differences in characteristics of glucose intolerance between patients with NAFLD and chronic hepatitis C as determined by CGMS

**DOI:** 10.1038/s41598-017-09256-4

**Published:** 2017-08-31

**Authors:** Tsunehiro Ochi, Takumi Kawaguchi, Takashi Nakahara, Masafumi Ono, Shuhei Noguchi, Yuichi Koshiyama, Kensuke Munekage, Eisuke Murakami, Akira Hiramatsu, Mitsunari Ogasawara, Akira Hirose, Hiroshi Mizuta, Kosei Masuda, Nobuto Okamoto, Narufumi Suganuma, Kazuaki Chayama, Masahiro Yamaguchi, Takuji Torimura, Toshiji Saibara

**Affiliations:** 1 Departments of Gastroenterology and Hepatology, Kochi Medical School, Kochi, Japan; 2 Departments of Physiology, Kochi Medical School, Kochi, Japan; 3 Division of Gastroenterology, Department of Medicine, Kurume University School of Medicine, Kurume, Japan; 4 Department of Gastroenterology and Metabolism, Division of Frontier, Medical Science, Programs for Biomedical Research Graduate School of Biomedical Science, Hiroshima University, Hiroshima, Japan; 5 Departments of Environmental Medicine, Kochi Medical School, Kochi, Japan

## Abstract

Glucose intolerance frequently develops in accordance with the progression of chronic liver disease. However, differences in the characteristics of glucose intolerance between patients with nonalcoholic fatty liver disease (NAFLD) and those with chronic hepatitis C (C-CH) remain incompletely understood. To clarify these differences, patients with NAFLD (n = 37) and C-CH (n = 40) were evaluated with a continuous glucose monitoring system (CGMS). In the patients with NAFLD, Maximum blood glucose concentration and blood glucose swings were significantly correlated with hepatic fibrosis markers. In the patients with C-CH, however, those two CGMS parameters were negatively correlated with the serum albumin (ALB) concentration. Furthermore, in the patients with C-CH with an ALB concentration of ≤4.0 g/dl, those two CGMS parameters were negatively correlated with the ALB concentration with greater statistical significance. In conclusion, obvious differences in the characteristics of glucose intolerance between patients with NAFLD and those with C-CH were clarified. In patients with NAFLD, glucose intolerance gradually progressed in accordance with the progression of hepatic fibrosis. In those with C-CH, glucose intolerance suddenly developed upon the appearance of hypoalbuminaemia.

## Introduction

Nonalcoholic fatty liver disease (NAFLD) (which includes a wide range of conditions from non-alcoholic fatty liver to nonalcoholic steatohepatitis [NASH]) and chronic hepatitis C (C-CH) are major causes of liver cirrhosis (LC) and hepatocellular carcinoma^1–3^. NAFLD/NASH is considered to be a hepatic manifestation of metabolic syndrome with impaired glucose tolerance^4, 5^. The number of patients with NAFLD/NASH has increased in accordance with the rising prevalence of obesity and type 2 diabetes mellitus (T2DM) worldwide^6, 7^. T2DM is considered to be associated with the progression of NAFLD/NASH^8, 9^. However, the presence of NAFLD/NASH is also considered to be associated with the development of T2DM^10^. C-CH is reportedly an important predictive factor for the development of T2DM^11, 12^. In addition, the prevalence of T2DM is remarkably higher in patients with C-CH than in those with chronic hepatitis B^11^.

T2DM is an independent risk factor for the development of chronic liver disease and hepatocellular carcinoma^13^, and chronic liver disease is a major cause of death in patients with DM^14, 15^. Furthermore, impaired glucose tolerance is frequently observed in patients with an advanced stage of chronic liver disease, which includes LC. Most patients with LC have insulin-resistant glucose intolerance (60–80%) and DM (about 20%)^16^. Proper control of insulin and glucose is important because inadequate control of blood glucose is associated with a poor prognosis in patients with LC^17^. Namely, chronic liver diseases are closely related to impaired glucose tolerance.

Continuous glucose monitoring systems (CGMSs) are useful tools with which to detect postprandial hyperglycaemia^18^, 24-hour glycaemic variability, and episodic hypoglycaemia while sleeping in patients with DM^19, 20^. A CGMS can be used to evaluate the minimum and maximum blood glucose concentrations, blood glucose swings (BGS) (Δmin–max blood glucose), average median blood glucose concentration, average blood glucose concentration, average standard deviation, duration of a blood glucose concentration of ≥180 mg/dl, and duration of a blood glucose concentration of <70 mg/dl. In particular, we previously clarified that blood glucose variability and hyperglycaemia are very important predictive factors for glucose impairment and hepatic fibrosis in patients with NAFLD^21^. Additionally, postprandial hyperglycaemia, postprandial hypoglycaemia, and glycaemic variability can reportedly cause atherosclerosis by oxidative stress and inflammatory cytokines^22–24^. Oxidative stress is one of the most important predictive factors for inflammation and progression of hepatic fibrosis in patients with NAFLD^25, 26^.

To inhibit the progression of glucose intolerance in patients with NAFLD and C-CH, it is important to clarify the differences in predictive factors for progression of glucose intolerance between these patients. However, differences in the characteristics of hyperglycaemia, hypoglycaemia, glycaemic variability, and the clinical features of glucose intolerance between patients with NAFLD and those with C-CH remain incompletely understood.

Therefore, we investigated differences in the characteristics of glucose intolerance between patients with NAFLD and C-CH using a CGMS.

## Results

Comparisons of the clinical and physiological data of patients with NAFLD and C-CH are shown in Table 1. Patients with C-CH were older than those with NAFLD. Hepatic fibrosis markers (type IV collagen 7S, type III procollagen N-peptide [P-3-P], and fibrosis-4 [FIB-4] index) and the prothrombin time (international normalised ratio [INR]) were significantly higher and the platelet (PLT) count and serum albumin (ALB) concentration were lower in patients with C-CH than in those with NAFLD. Although the fasting immunoreactive insulin concentration (f-IRI) and homeostatic model assessment of insulin resistance [HOMA-IR] were significantly higher in patients with NAFLD than in those with C-CH, other parameters of glucose intolerance (glycated haemoglobin [HbA1c], and 1,5-anhydroglucitol[1,5-AG]) were not significantly different between the two groups.

**Table 1:** Clinical and physiological characteristics of patients with NAFLD or C-CH.

	NAFLD (=37)	C-CH (=40)	P value
Gender (F/M)	19/18	17/23	ns
Age (yo)	54.4 ± 16.2	69.5 ± 9.5	<0.001
BMI (Kg/m^2^)	29.6 ± 7.3	24.0 ± 3.7	<0.001
FBS (mg/dl)	11.5 ± 29.8	113.4 ± 41.9	ns
f-IRI (μU/ml)	19.4 ± 17.1	11.1 ± 6.9	<0.05
HOMA-IR	5.15 ± 4.70	3.18 ± 2.22	<0.05
HbA1c (%)	6.3 ± 1.6	6.3 ± 1.3	ns
1,5-AG (μg/ml)	15.3 ± 7.6	15.3 ± 10.3	ns
AST (IU/L)	64.9 ± 26.7	55.8 ± 26.0	ns
ALT (IU/L)	89.7 ± 49.4	43.7 ± 22.9	<0.001
ALP (IU/L)	275.1 ± 69.8	387.8 ± 163.1	<0.001
GGT (IU/L)	101.5 ± 82.9	68.3 ± 75.7	ns
T-Bil (mg/dl)	0.93 ± 0.41	1.05 ± 0.85	ns
TP (g/dl)	7.4 ± 0.5	7.2 ± 0.7	ns
ALB (g/dl)	4.4 ± 0.5	3.5 ± 0.7	<0.001
T-CHO (mg/dl)	201.7 ± 33.4	149.9 ± 33.1	<0.001
TG (mg/dl)	152.5 ± 71.9	103.7 ± 50.1	<0.005
RBC (×10^4^/μl)	460.0 ± 52.1	384.3 ± 54.2	<0.001
Hb (g/dl)	14.3 ± 1.4	12.4 ± 1.7	<0.001
PLT (×10^4^/μl)	18.9 ± 6.9	12.8 ± 6.6	<0.001
WBC (×10^3^/μl)	6.0 ± 1.5	5.0 ± 2.0	<0.05
IV collagen 7S (ng/ml)	5.1 ± 2.1	8.8 ± 3.7	<0.001
P-3-P (U/ml)	0.7 ± 0.3	1.2 ± 0.3	<0.001
FIB-4 index	2.69 ± 2.07	6.69 ± 5.61	<0.001
PT (INR)	1.04 ± 0.08	1.16 ± 0.22	<0.005

The CGMS parameters between the patients with NAFLD and C-CH are shown in Table 2. Although the average minimum blood glucose and average blood glucose concentration in patients with NAFLD were significantly lower than that in patients with C-CH, no differences were found in other parameters (maximum blood glucose, BGS, average median blood glucose, average standard deviation, blood glucose of ≥180 mg/dl [% and time], and blood glucose of <70 mg/dl [% and time]) between the two groups of patients.

**Table 2:** Parameters of CGMS in patients with NAFLD or C-CH.

Variable	NAFLD (=37)	C-CH (=40)	P value
Minimum blood glucose (mg/dl)	78.9 ± 19.0	93.4 ± 26.3	<0.01
Maximum blood glucose (mg/dl)	205.2 ± 55.3	222.8 ± 60.6	ns
Blood glucose swings (mg/dl)	123.0 ± 58.3	129.4 ± 54.1	ns
Average median blood glucose (mg/dl)	125.8 ± 26.4	139.1 ± 35.7	ns
Average blood glucose (mg/dl)	128.3 ± 25.4	143.2 ± 33.8	<0.05
Average standard deviation (mg/dl)	28.4 ± 14.7	30.7 ± 15.3	ns
Blood glucose ≧ 180 mg/dl time (**%**)	11.1 ± 15.2	19.9 ± 25.7	ns
Blood glucose ≧ 180 mg/dl time (minutes)	174.4 ± 225.1	287.8 ± 371.4	ns
Blood glucose <70 mg/dl time (**%**)	1.9 ± 3.8	0.7 ± 2.4	ns
Blood glucose <70 mg/dl time (minutes)	27.8 ± 57.2	10.6 ± 34.8	ns

Next, we examined the number of patients with hyperglycaemia (maximum blood glucose of ≥180 mg/dl), hypoglycaemia (minimum blood glucose of <70 mg/dl), and excessive glycaemic variability (BGS of ≥110 mg/dl) (Supplemental Table 1). Hyperglycaemia more frequently occurred in patients with C-CH (n = 29) than in those with NAFLD (n = 24). In contrast, hypoglycaemia more frequently occurred in patients with NAFLD (n = 11) than in those with C-CH (n = 6). However, there was no difference in the frequency of excessive glycaemic variability between patients with NAFLD (n = 18) and those with C-CH (n = 24).

We also examined the clinical and physiological parameters of the patients with hyperglycaemia, hypoglycaemia, and excessive glycaemic variability between those with NAFLD and C-CH (Tables 3 and 4). Among patients with NAFLD, Age, FBS, HbA1c and type IV collagen 7S were higher in patients with hyperglycaemia, total bilirubin and P-3-P were higher and ALT and ALB were lower in patients with hypoglycaemia, and ALB and PLT count were lower and hepatic fibrosis markers (type IV collagen 7S, P-3-P, and FIB-4 index) were higher in patients with excessive glycaemic variability (Table 3). Among patients with C-CH, 1,5-AG, total protein (TP), ALB and total cholesterol were lower in patients with hyperglycaemia, while HbA1c was lower in patients with hypoglycaemia, and TP, ALB and total cholesterol were lower in patients with excessive glycaemic variability (Table 4).

**Table 3:** Clinical and physiological characteristics of patients with hyperglycaemia, hypoglycaemia, and excessive glycaemic variability among patients with NAFLD.

NAFLD	Hyperglycaemia			Hypoglycaemia			Excessive glycaemic variability		
	(+)	(−)	P value	(+)	(−)	P value	(+)	(−)	P value
Gender (F/M)	13/11	6/7	ns	6/5	13/13	ns	9/9	10/9	ns
Age (yo)	58.5 ± 12.9	46.7 ± 18.6	<0.05	53.9 ± 14.2	54.6 ± 17.0	ns	58.8 ± 9.6	50.2 ± 19.7	ns
BMI (Kg/m^2^)	28.6 ± 7.6	31.3 ± 6.2	ns	31.7 ± 9.9	28.7 ± 5.6	ns	28.9 ± 8.7	30.3 ± 5.5	ns
FBS (mg/dl)	118.0 ± 33.9	96.7 ± 10.5	<0.05	106.2 ± 32.1	112.3 ± 28.6	ns	112.0 ± 28.2	109.1 ± 31.2	ns
f-IRI (μU/ml)	18.4 ± 13.9	21.0 ± 21.3	ns	22.8 ± 17.9	18.0 ± 16.5	ns	19.2 ± 15.1	19.5 ± 18.6	ns
HOMA-IR	5.07 ± 3.80	5.27 ± 5.91	ns	5.55 ± 4.86	4.98 ± 4.62	ns	4.99 ± 4.10	5.28 ± 5.14	ns
HbA1c (%)	6.7 ± 1.8	5.5 ± 0.4	<0.05	6.0 ± 1.2	6.4 ± 1.7	ns	6.3 ± 1.3	6.2 ± 1.8	ns
1,5-AG (μg/ml)	13.4 ± 7.5	18.9 ± 6.6	ns	13.1 ± 5.4	15.9 ± 8.1	ns	14.9 ± 7.9	15.7 ± 7.4	ns
AST (IU/L)	69.3 ± 27.5	56.8 ± 23.0	ns	58.2 ± 26.5	67.8 ± 26.3	ns	64.7 ± 23.4	65.1 ± 29.4	ns
ALT (IU/L)	93.3 ± 52.0	83.2 ± 43.5	ns	62.7 ± 29.1	101.2 ± 51.8	<0.05	75.7 ± 36.0	103.0 ± 56.3	ns
ALP (IU/L)	285.1 ± 69.3	256.5 ± 67.0	ns	269.9 ± 72.3	277.2 ± 68.6	ns	285.3 ± 77.7	265.4 ± 59.8	ns
GGT (IU/L)	107.1 ± 80.9	91.0 ± 84.0	ns	104.1 ± 85.3	100.3 ± 81.1	ns	110.0 ± 86.5	93.4 ± 77.4	ns
T-Bil (mg/dl)	0.91 ± 0.41	0.98 ± 0.40	ns	1.15 ± 0.48	0.85 ± 0.34	<0.05	1.02 ± 0.48	0.85 ± 0.31	ns
TP (g/dl)	7.3 ± 0.5	7.5 ± 0.3	ns	7.2 ± 0.5	7.5 ± 0.4	ns	7.3 ± 0.5	7.5 ± 0.3	ns
ALB (g/dl)	4.3 ± 0.5	4.6 ± 0.3	ns	4.2 ± 0.4	4.5 ± 0.4	<0.05	4.2 ± 0.5	4.6 ± 0.3	<0.05
T-CHO (mg/dl)	196.8 ± 31.7	210.8 ± 34.4	ns	202.3 ± 39.0	201.5 ± 30.7	ns	192.6 ± 33.8	210.4 ± 30.5	ns
TG (mg/dl)	159.5 ± 61.5	140.6 ± 85.3	ns	128.8 ± 46.2	162.0 ± 77.8	ns	157.6 ± 65.8	148.2 ± 76.3	ns
RBC (×10^4^/μl)	457.1 ± 48.4	465.5 ± 58.0	ns	448.4 ± 52.2	465.0 ± 51.3	ns	453.6 ± 45.9	466.1 ± 56.7	ns
Hb (g/dl)	14.1 ± 1.3	14.6 ± 1.5	ns	14.1 ± 1.2	14.4 ± 1.5	ns	14.2 ± 1.3	14.4 ± 1.5	ns
PLT (×10^4^/μl)	18.5 ± 7.0	19.7 ± 6.8	ns	15.5 ± 6.1	20.4 ± 6.8	ns	15.7 ± 6.4	22.0 ± 6.0	<0.005
WBC (×10^3^/μl)	6.2 ± 1.4	5.7 ± 1.7	ns	5.4 ± 1.5	6.3 ± 1.5	ns	5.7 ± 1.4	6.3 ± 1.6	ns
IV collagen 7S (ng/ml)	5.8 ± 2.1	3.9 ± 1.4	<0.01	5.9 ± 2.2	4.8 ± 1.9	ns	6.1 ± 2.1	4.2 ± 1.6	<0.01
P-3-P (U/ml)	0.8 ± 0.3	0.6 ± 0.2	ns	0.9 ± 0.3	0.7 ± 0.2	<0.05	0.8 ± 0.3	0.6 ± 0.2	<0.05
FIB-4 index	3.00 ± 2.14	2.10 ± 1.80	ns	3.53 ± 2.58	2.33 ± 1.69	ns	3.62 ± 2.29	1.80 ± 1.34	<0.01
PT (INR)	1.05 ± 0.09	1.04 ± 0.06	ns	1.09 ± 0.13	1.03 ± 0.05	ns	1.06 ± 0.10	1.03 ± 0.05	ns

Hyperglycaemia: maximum blood glucose of ≥180 mg/dl; hypoglycaemia: minimum blood glucose of <70 mg/dl; excessive glycaemic variability: blood glucose swings of ≥110 mg/dl.

**Table 4:** Clinical and physiological characteristics of patients with hyperglycaemia, hypoglycaemia, and excessive glycaemic variability among patients with C-CH.

C-CH	Hyperglycaemia			Hypoglycaemia			Excessive glycaemic variability		
	(+)	(−)	P value	(+)	(−)	P value	(+)	(−)	P value
Gender (F/M)	11/18	6/5	ns	4/2	13/21	ns	8/16	9/7	ns
Age (yo)	69.6 ± 9.2	69.2 ± 10.3	ns	73.8 ± 6.7	68.7 ± 9.7	ns	71.3 ± 8.0	66.7 ± 10.8	ns
BMI (Kg/m^2^)	23.6 ± 2.8	25.1 ± 5.3	ns	25.3 ± 4.4	23.8 ± 3.6	ns	23.5 ± 2.9	24.8 ± 4.6	ns
FBS (mg/dl)	119.2 ± 46.7	98.1 ± 17.8	ns	87.0 ± 4.8	118.1 ± 43.8	ns	114.7 ± 44.5	111.5 ± 37.6	ns
f-IRI (μU/ml)	11.8 ± 7.5	9.3 ± 4.2	ns	10.5 ± 6.3	11.3 ± 7.0	ns	11.5 ± 7.6	10.6 ± 5.6	ns
HOMA-IR	3.50 ± 2.40	2.29 ± 1.22	ns	2.24 ± 1.31	3.38 ± 2.32	ns	3.35 ± 2.54	2.94 ± 1.63	ns
HbA1c (%)	6.5 ± 1.3	5.6 ± 1.1	ns	5.2 ± 0.5	6.5 ± 1.3	<0.05	6.6 ± 1.3	5.8 ± 1.1	ns
1,5-AG (μg/ml)	13.5 ± 9.3	30.2 ± 4.6	<0.05	16.9 ± 10.5	15.2 ± 10.3	ns	13.3 ± 8.4	20.6 ± 12.6	ns
AST (IU/L)	57.3 ± 27.1	51.7 ± 22.1	ns	60.7 ± 31.3	54.9 ± 24.8	ns	59.5 ± 28.3	50.2 ± 20.8	ns
ALT (IU/L)	43.9 ± 24.2	43.1 ± 19.2	ns	32.5 ± 11.3	45.6 ± 23.9	ns	46.3 ± 25.6	39.7 ± 17.5	ns
ALP (IU/L)	380.5 ± 164.1	409.1 ± 158.3	ns	362.2 ± 160.9	391.6 ± 163.1	ns	360.6 ± 160.8	431.5 ± 157.2	ns
GGT (IU/L)	65.8 ± 48.2	74.8 ± 120.9	ns	97.8 ± 159.4	63.0 ± 45.5	ns	72.8 ± 50.1	61.5 ± 102.3	ns
T-Bil (mg/dl)	1.11 ± 0.97	0.87 ± 0.38	ns	0.96 ± 0.43	1.06 ± 0.91	ns	0.88 ± 0.36	1.30 ± 1.24	ns
TP (g/dl)	7.0 ± 0.7	7.6 ± 0.6	<0.05	7.5 ± 1.0	7.1 ± 0.6	ns	7.0 ± 0.7	7.4 ± 0.6	<0.05
ALB (g/dl)	3.3 ± 0.7	4.1 ± 0.5	<0.005	3.6 ± 0.6	3.5 ± 0.8	ns	3.3 ± 0.8	3.8 ± 0.6	<0.05
T-CHO (mg/dl)	141.2 ± 29.6	171.2 ± 31.7	<0.05	156.0 ± 20.7	148.7 ± 34.8	ns	140.4 ± 26.0	162.9 ± 37.1	<0.05
TG (mg/dl)	98.1 ± 43.8	118.4 ± 61.5	ns	94.3 ± 22.4	104.8 ± 52.3	ns	103.6 ± 44.5	103.8 ± 56.3	ns
RBC (×10^4^/μl)	376.9 ± 51.6	403.7 ± 56.0	ns	350.8 ± 20.8	390.2 ± 56.1	ns	381.1 ± 46.1	389.1 ± 64.2	ns
Hb (g/dl)	12.2 ± 1.7	13.0 ± 1.5	ns	11.3 ± 0.8	12.6 ± 1.7	ns	12.4 ± 1.6	12.5 ± 1.8	ns
PLT (×10^4^/μl)	11.8 ± 7.0	15.5 ± 4.7	ns	13.4 ± 5.4	12.7 ± 6.8	ns	12.8 ± 7.1	12.9 ± 5.9	ns
WBC (×10^3^/μl)	4.8 ± 2.0	5.6 ± 1.9	ns	4.3 ± 1.3	5.1 ± 2.1	ns	5.0 ± 2.1	5.0 ± 2.0	ns
IV collagen 7S (ng/ml)	9.6 ± 3.8	6.9 ± 2.7	ns	8.0 ± 2.0	9.0 ± 4.0	ns	9.1 ± 3.2	8.4 ± 4.2	ns
P-3-P (U/ml)	1.2 ± 0.3	1.1 ± 0.1	ns	1.2 ± 0.1	1.2 ± 0.3	ns	1.2 ± 0.3	1.2 ± 0.2	ns
FIB-4 index	7.52 ± 6.15	4.40 ± 2.79	ns	7.15 ± 5.44	6.58 ± 5.64	ns	6.69 ± 4.36	6.63 ± 7.08	ns
PT (INR)	1.17 ± 0.23	1.12 ± 0.16	ns	1.22 ± 0.22	1.15 ± 0.21	ns	1.14 ± 0.16	1.18 ± 0.28	ns

Hyperglycaemia: maximum blood glucose of ≥80 mg/dl; hypoglycaemia: minimum blood glucose of <70 mg/dl; excessive glycaemic variability: blood glucose swings of ≥110 mg/dl.

### Correlations of clinical and physiological parameters with maximum blood glucose, minimum blood glucose, and BGS in patients with NAFLD and C-CH

**Figure 1:**
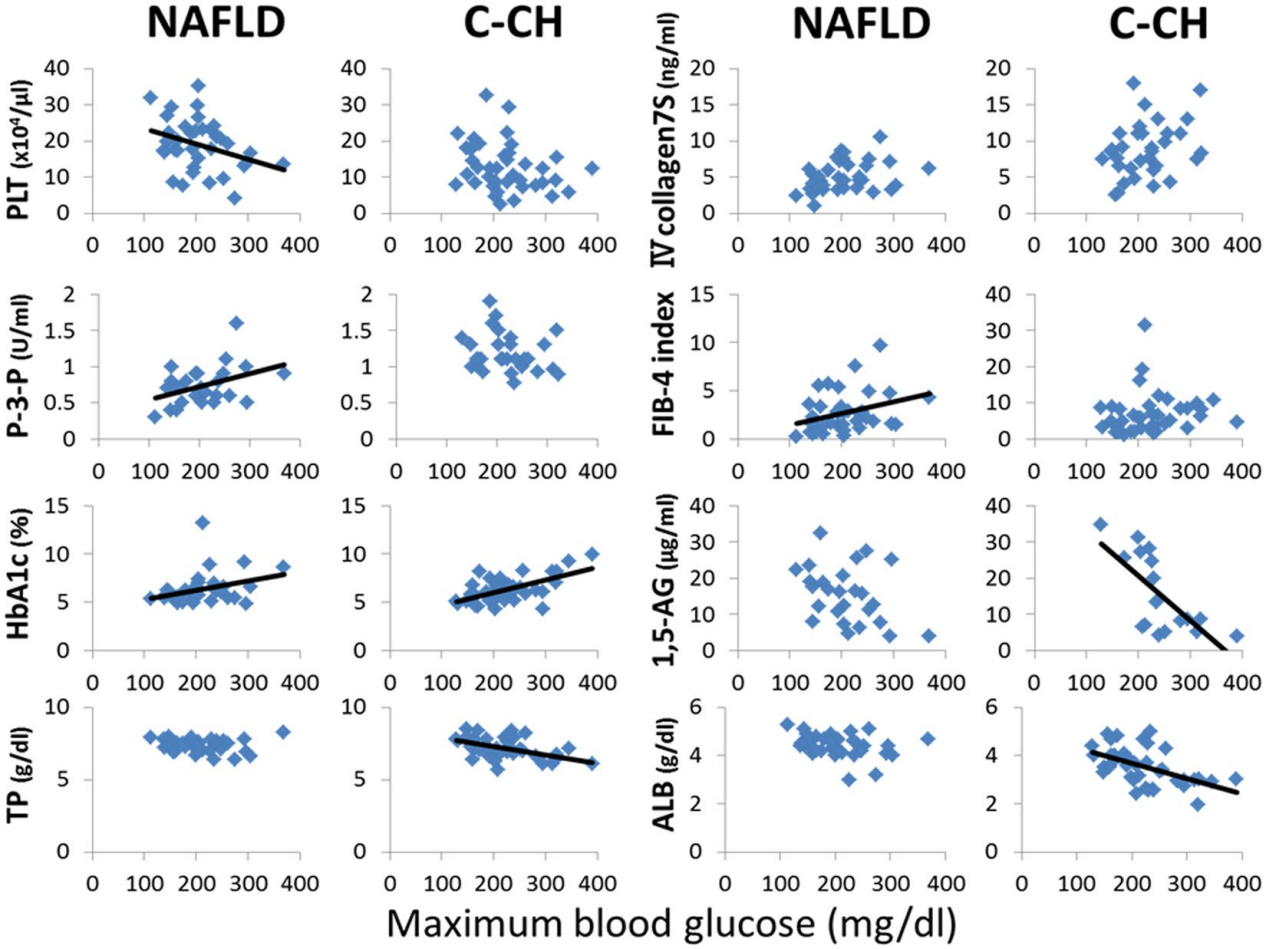
Relationship between maximum blood glucose and various parameters in patients with NAFLD or C-CH. The maximum blood glucose was correlated with the fibrosis markers PLT count (r = −0.3388, P < 0.05), P-3-P (r = 0.4051, P < 0.05) and FIB-4 index (r = 0.3253, P < 0.05) in patients with NAFLD (n = 37) and with TP (r = −0.4749, P < 0.005), ALB (r = −0.5147, P < 0.001), HbA1c (r = 0.6027, P < 0.001), and 1,5-AG (r = −0.7020, P < 0.005) but not with fibrosis markers in patients with C-CH (n = 40).

**Figure 2:**
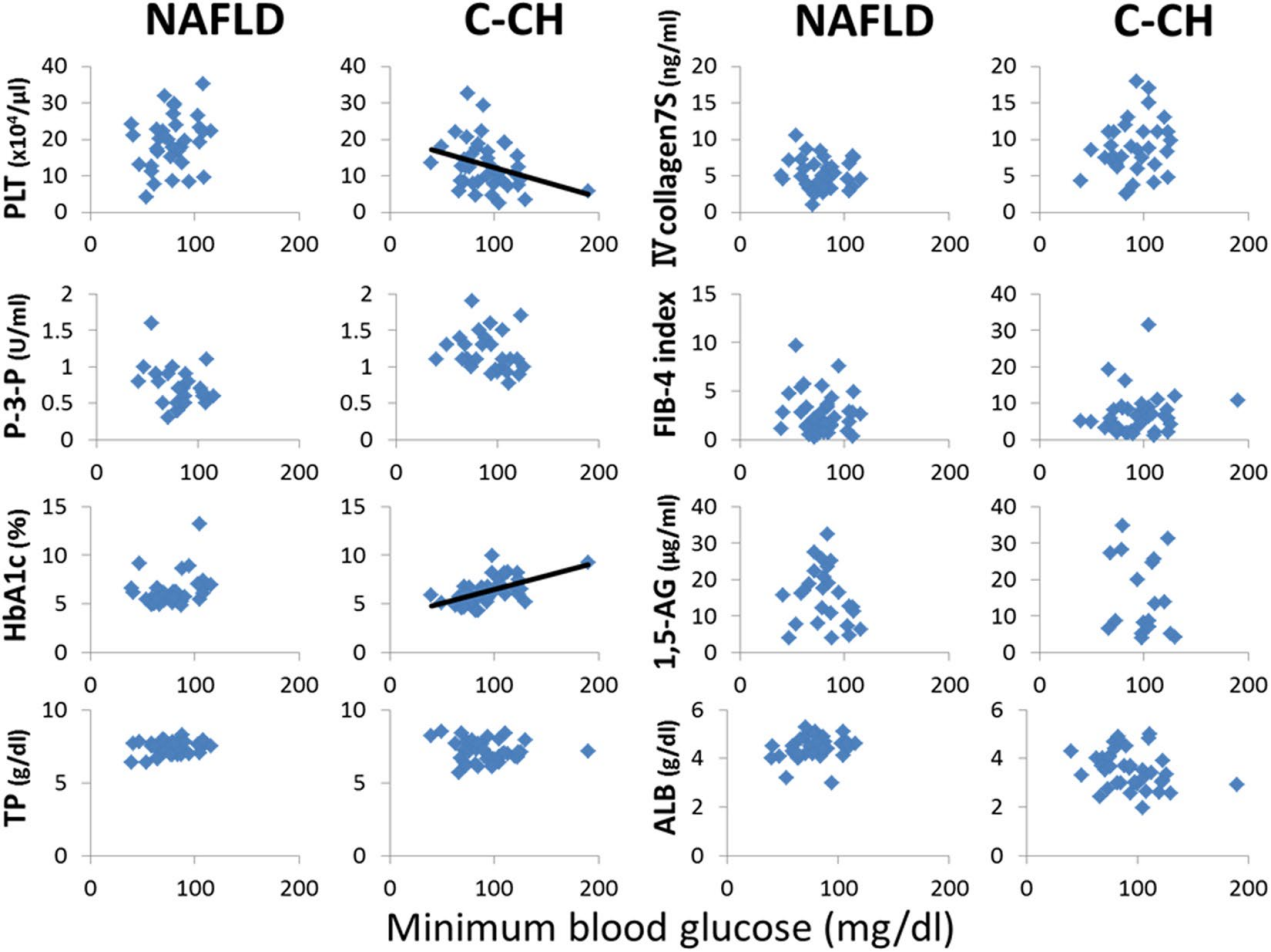
Relationship between minimum blood glucose and various parameters in patients with NAFLD or C-CH. The minimum blood glucose was correlated with the PLT count (r = −0.3209, P < 0.05) and HbA1c (r = −0.5739, P < 0.001) in patients with C-CH (n = 40), but was not correlated with any parameters of liver function tests in patients with NAFLD (n = 37).

**Figure 3:**
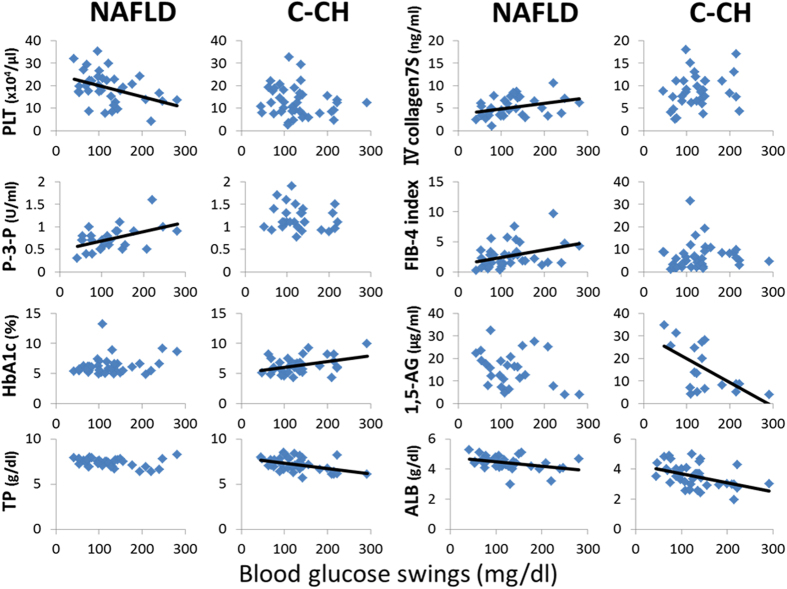
Relationship between BGS and various parameters in patients with NAFLD and C-CH. BGS were correlated with ALB (r = −0.3709, P < 0.05) and fibrosis markers such as the PLT count (r = −0.4114, P < 0.05), type IV collagen 7S (r = 0.3556, P < 0.05), P-3-P (r = 0.4796, P < 0.05), and the FIB-4 index (r = 0.3510, P < 0.05) in patients with NAFLD (n = 37) and with TP (r = −0.4574, P < 0.005), ALB (r = −0.4341, P < 0.01), HbA1c (r = 0.3963, P < 0.05), and 1,5-AG (r = −0.6193, P < 0.01) but not fibrosis markers in patients with C-CH (n = 40).

We next focused on the correlations of clinical and physiological parameters (PLT count, hepatic fibrosis markers [type IV collagen 7S, P-3-P, and FIB-4 index], HbA1c, 1,5-AG, TP, and ALB) with the maximum blood glucose, minimum blood glucose, and BGS in patients with NAFLD and C-CH (Figs 1–3). The maximum blood glucose was correlated with PLT count (r = −0.3388, P < 0.05), P-3-P (r = 0.4051, P < 0.05), FIB-4 index (r = 0.3253, P < 0.05) and HbA1c (r = 0.3425, P < 0.05) in patients with NAFLD (Fig. 1). In patients with C-CH, however, the maximum blood glucose was correlated with TP (r = −0.4749, P < 0.005), ALB (r = −0.5147, P < 0.001), HbA1c (r = 0.6027, P < 0.001), and 1,5-AG (r = −0.7020, P < 0.005) but not with the hepatic fibrosis markers. Analysis of the relationship between the minimum blood glucose and clinical parameters showed that the PLT count (r = −0.3209, P < 0.05) and HbA1c (r = −0.5739, P < 0.001) were correlated with the minimum blood glucose in patients with C-CH (Fig. 2). In contrast, no parameters were correlated with the minimum blood glucose in patients with NAFLD. Interestingly, analysis of the relationship between BGS and various parameters showed that the factors for these correlations were different between patients with NAFLD and those with C-CH (Fig. 3). In patients with NAFLD, BGS were significantly correlated with ALB (r = −0.3709, P < 0.05) and the following hepatic fibrosis markers: type IV collagen 7S (r = 0.3556, P < 0.05), P-3-P (r = 0.4796, P < 0.05), the PLT count (r = −0.4114, P < 0.05), and the FIB-4 index (r = 0.3510, P < 0.05). In contrast, in patients with C-CH, BGS were significantly correlated with TP (r = −0.4574, P < 0.005), ALB (r = −0.4341, P < 0.01), HbA1c (r = 0.3963, P < 0.05), and 1,5-AG (r = −0.6193, P < 0.01) but not with the hepatic fibrosis markers.

### Association of maximum blood glucose and BGS with ALB reduction in advanced stage of LC in patients with C-CH

**Figure 4:**
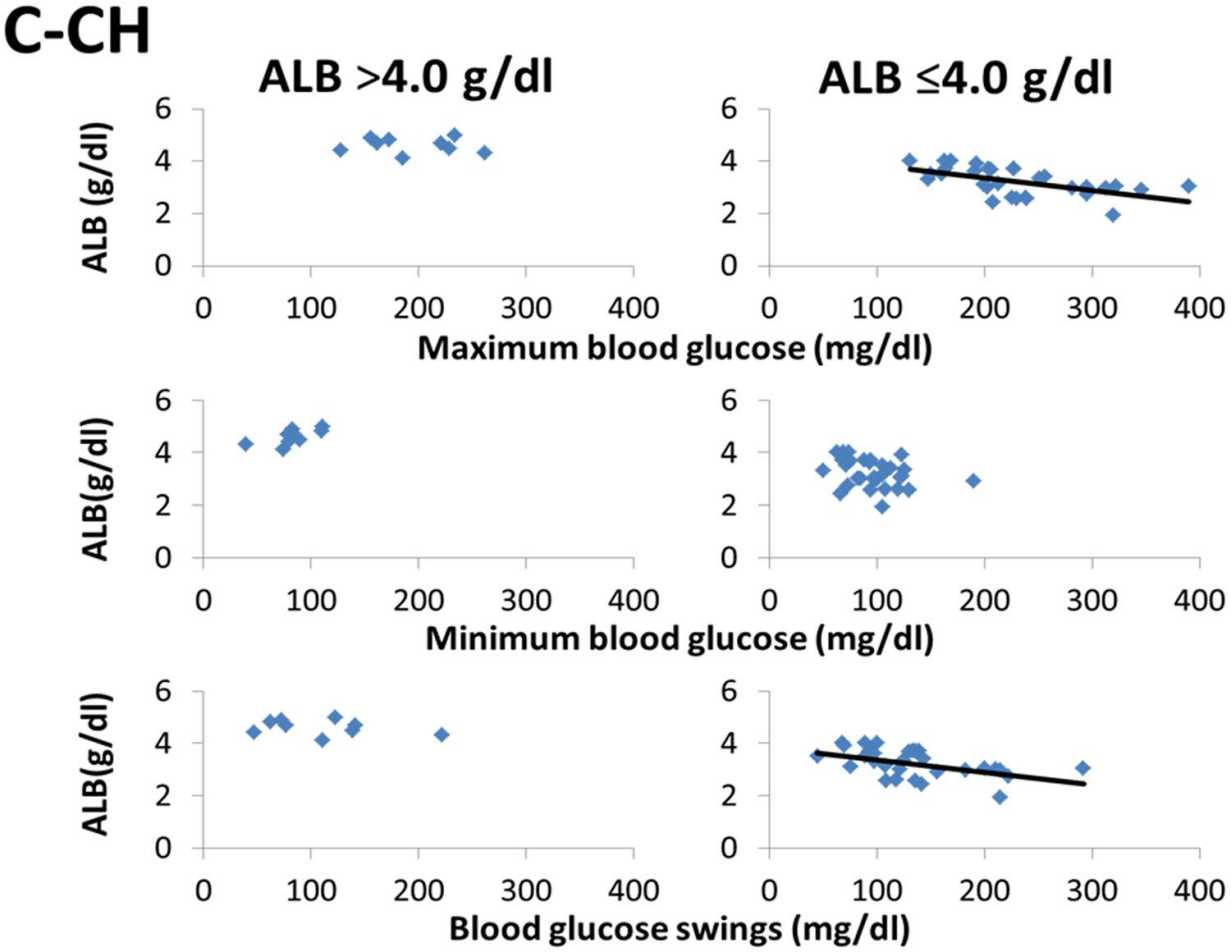
Relationship between blood glucose and ALB in patients with C-CH with ALB of ≤4.0 or >4.0 g/dl. In patients with C-CH with ALB of ≤4.0 g/dl (n = 31), the maximum blood glucose (r = −0.5722, P < 0.001) and BGS (r = −0.5006, P < 0.005) were correlated with the ALB concentration, but these correlations were not found in patients with ALB of >4.0 g/dl (n = 9).

We further investigated the details of the correlation between ALB and the maximum blood glucose, minimum blood glucose, and BGS in patients with C-CH with higher and lower ALB concentrations (>4.0 and ≤4.0 g/dl, respectively). We performed this investigation because hypoalbuminaemia is reportedly closely related to the development of glucose intolerance in patients with hepatitis virus-related LC^27^. BGS (r = −0.5006, P < 0.005) and the maximum blood glucose (r = −0.5722, P < 0.001) were negatively correlated with ALB in the patients with an ALB concentration of ≤4.0 g/dl (n = 31) (Fig. 4). In contract, neither BGS (r = −0.3230, P = ns) nor the maximum blood glucose (r = −0.0708, P = ns) were correlated with ALB in the patients with an ALB concentration of >4.0 g/dl (n = 9).

## Discussion

This is the first study to clarify the differences in the characteristics of glucose intolerance between patients with NAFLD and those with C-CH. In patients with NAFLD, hyperglycaemia and excessive glycaemic variability gradually progressed in accordance with the progression of hepatic fibrosis from the early stage of chronic liver disease. In patients with C-CH, however, hyperglycaemia and glycaemic variability suddenly progressed when hypoalbuminaemia appeared.

All clinical characteristics of glucose intolerance in both groups, with the exception of insulin resistance, were quite similar as shown by blood testing and use of the CGMS, although patients with C-CH had a more advanced stage of chronic liver disease than did patients with NAFLD. However, each characteristic of glucose intolerance in both groups was clarified in detail. Hypoglycaemia (blood glucose of ≤70 mg/dl) more frequently occurs in patients with NAFLD than in those with C-CH. Conversely, hyperglycaemia (blood glucose of ≥180 mg/dl) more frequently occurs in patients with C-CH than in those with NAFLD (Table S1).

In the patients with NAFLD, remarkable differences in the characteristics of the laboratory data were found between patients with and without excessive glycaemic variability (BGS of ≥110 and <110 mg/dl, respectively) (Table 3). Fibrosis markers (Type IV collagen 7S, P-3-P, and the FIB-4 index) were significantly higher in patients with than without excessive glycaemic variability. Additionally, the PLT count, which is also a hepatic fibrosis marker^28^, was remarkably lower in patients with excessive glycaemic variability. Furthermore, BGS were significantly correlated with the following hepatic fibrosis markers: type IV collagen 7S, P-3-P, PLT count, and FIB-4 index (Fig. 3). These findings are agreement with our previous report in which glycaemic variability was an independent predictive factor for the development of hepatic fibrosis in patients with NAFLD^26^. Thus, the development of hepatic fibrosis appears to be closely related to the progression of glucose impairment and glycaemic variability. In other words, these characteristic glucose intolerances gradually develop in accordance with the progression of hepatic fibrosis beginning in the early stage of chronic liver disease in patients with NAFLD.

In patients with C-CH, however, TP and ALB were lower in patients with than without hyperglycaemia (Table 4). In addition, the maximum blood glucose was well negatively correlated with the TP and ALB concentrations (Fig. 1). These results indicate that advanced liver disease might be closely related to hyperglycaemia. Similarly TP and ALB were lower in patients with than without excessive glycaemic variability (BGS of ≥110 and <110 mg/dl, respectively) (Table 4). BGS were also significantly negatively correlated with the TP and ALB concentrations, but not with hepatic fibrosis markers, unlike in patients with NAFLD (Fig. 3). It is well known that hypoalbuminaemia indicates the presence of advanced chronic liver disease, such as LC. In contrast, elevation of hepatic fibrosis markers does not always indicate the presence of advanced chronic liver disease, but instead indicates progression from early stages of chronic liver diseases. Thus, the progression of glucose impairment and glycaemic variability were closely related to the presence of advanced liver diseases in patients with C-CH. Previous studies have shown that hypoalbuminaemia is an important predictive factor for T2DM in patients with hepatitis virus-related LC^27, 29^. Therefore, we further investigated the glucose intolerance in patients with C-CH who did and did not have hypoalbuminaemia (ALB of ≤4.0 and >4.0 g/dl, respectively) (Fig. 4). The serum ALB concentration was significantly negatively correlated with the maximum blood glucose and BGS in patients with an ALB concentration of ≤4.0 g/dl. However, no correlations were found in patients with an ALB concentration of >4.0 g/dl. These findings suggest that glucose intolerance, hyperglycaemia, and glycaemic variability might suddenly progress when hypoalbuminaemia appears in patients with C-CH. The cut-off levels of ALB and hepatic fibrosis markers could not be established in patients with NAFLD, unlike in the analysis of patients with C-CH (data not shown). These results may indicate that glucose intolerance gradually develops in accordance with the progression of hepatic fibrosis beginning in the early stage of chronic liver disease in patients with NAFLD, unlike in those with C-CH.

The limitation of this study might be the small number of patients. Although the clinical characteristics of glucose intolerance as indicated by blood tests and the CGMS were quite similar between the two groups in this study, patients with C-CH had more advanced stages of chronic liver disease than did patients with NAFLD. Furthermore, although the diagnoses of NAFLD were obtained by liver biopsy, biopsies were not performed in patients with C-CH. Therefore, histological analysis could not be carried out.

In conclusion, this is the first report of the differences in the characteristics of glucose intolerance between patients with C-CH and those with NAFLD using a CGMS. Hypoglycaemia more frequently occurs in patients with NAFLD than in those with C-CH; in contrast, hyperglycaemia more frequently occurs in patients with C-CH than in those with NAFLD. In patients with NAFLD, hyperglycaemia and glycaemic variability gradually progress in accordance with the progression of hepatic fibrosis beginning in the early stage of chronic liver disease. Conversely, in patients with C-CH, hyperglycaemia and glycaemic variability suddenly progress when hypoalbuminaemia becomes evident. These different characteristics of glucose intolerance may be beneficial for the clinical management of patients with NAFLD and C-CH.

## Methods

### Patients

37 patients with biopsy-proven NAFLD (19 female, 18 male) and 40 patients with C-CH (17 female, 23 male) who underwent CGMS monitoring were enrolled in this study. Liver biopsies and thorough clinical evaluation were performed in all patients with NAFLD under written informed consent. All patients with NAFLD with known use of methotrexate, tamoxifen, corticosteroids, or alcohol in excess of 20 g per day as well as patients with other known causes of liver disease including viral hepatitis, hemochromatosis, Wilson’s disease, and autoimmune hepatitis were excluded from this study. In all patients with C-CH, the diagnosis was confirmed by hepatitis C virus RNA positivity and exclusion of other types of viral hepatitis and metabolic liver diseases, including fatty liver disease. None of the patients had received any anti-diabetic drugs or insulin. The study protocol conformed to the ethical guidelines of the 1975 Declaration of Helsinki^30^ and was approved by the Research Committee of Kochi Medical School, Kurume University and Hiroshima University.

### Clinical and laboratory evaluations

Venous blood samples were obtained the morning after a 12-hour overnight fast. Laboratory measurements in all patients included the serum concentrations of aspartate aminotransferase, alanine aminotransferase, gamma-glutamyl transpeptidase, lipids, total cholesterol, triglycerides, high-density lipoprotein cholesterol, low-density lipoprotein cholesterol, fasting plasma glucose, fasting immunoreactive insulin, creatinine, blood urea nitrogen, 1,5-AG, HbA1c, and fibrosis markers. These parameters were measured using standard clinical chemistry techniques in the laboratory section of Kochi Medical School Hospital, Kurume University Hospital and Hiroshima University Hospital. Insulin resistance was calculated by HOMA-IR using the following formula: HOMA-IR = fasting plasma insulin (μU/ml) × fasting plasma glucose (mg/dl)/405. Liver fibrosis was calculated by the FIB-4 index using the following formula: FIB-4 index = [age (years) × AST (IU/L)]/[PLT (×109/L) ×square root of ALT (IU/L)]^31^.

### CGMS

Continuous glucose levels in 37 patients with biopsy-proven NAFLD and 40 patients with C-CH were monitored by the CGMS System Gold (Medtronic MiniMed, Northridge, CA, USA). According to the operating guidelines, the CGMS was installed in the patients to monitor the glucose levels of interstitial fluid. The glucose sensor was inserted into the subcutaneous tissue of the abdomen at 3:00 to 4:00 PM and was monitored for 30 hours. Finger-stick blood glucose levels were checked to calibrate the first glucose value of the CGMS after 1 hour of initialisation. Glucose concentrations were determined at least four times per day with an automatic blood glucose meter (Glutest; Sanwa Kagaku Kenkyusho Co., Ltd., Nagoya, Japan). Meals were strictly standardised (1800 kcal/day of a standard diet) during the examination.

### Statistical analyses

Results are presented as mean ± standard deviation for quantitative data and as number or percentage for categorical or qualitative data. Statistical differences in quantitative data were determined using the Mann–Whitney U test or post-hoc test. Qualitative data were compared using the chi-square test. Correlations were calculated by Spearman’s rank correlation analysis. These statistical analyses were carried out using Small Stata 10.1 for Windows (StataCorp, College Station, TX, USA). Results were considered significant when the P value was < 0.05.

## Electronic supplementary material

Supplementary Information
