# Supplementary material for: Differences in characteristics of glucose intolerance between patients with NAFLD and chronic hepatitis C as determined by CGMS
Source: Sci Rep. 2017 Aug 31;7:10146. doi: 10.1038/s41598-017-09256-4 (PMC5579047; doi:10.1038/s41598-017-09256-4)
Supplement: Supplementary file 1 — Supplementary Information [file 41598_2017_9256_MOESM1_ESM.pdf]

Supplementary information.

Differences in characteristics of glucose intolerance between patients with NAFLD and chronic hepatitis C as determined by CGMS

Tsunehiro Ochi[1,2], Takumi Kawaguchi[4], Takashi Nakahara[5], Masafumi Ono[1], Shuhei Noguchi[3], Yuichi Koshiyama[1], Kensuke Munekage[1], Eisuke Murakami[5], Akira Hiramatsu[5], Mitsunari Ogasawara[1], Akira Hirose[1], Hiroshi Mizuta[1], Kosei Masuda[1], Nobuto Okamoto[1], Narufumi Suganuma[3], Kazuaki Chayama[5], Masahiro Yamaguchi[2], Takuji Torimura[4] and Toshiji Saibara[1]

Departments of [1]Gastroenterology and Hepatology, [2]Physiology, and [3]Environmental Medicine, Kochi Medical School, Kochi, Japan.
[4]Division of Gastroenterology, Department of Medicine, Kurume University School of Medicine, Kurume, Japan.
[5]Department of Gastroenterology and Metabolism, Division of Frontier Medical Science, Programs for Biomedical Research Graduate School of Biomedical Science, Hiroshima University, Hiroshima, Japan.

Table S1. Comparison of clinical and physiological characteristics between NAFLD and C-CH patients with hyperglycaemia, hypoglycaemia, and excessive glycaemic variability.

	Hyperglycaemia			Hypoglycaemia			Excessive glycaemic variability		
Disease	NAFLD	C-CH	P value	NAFLD	C-CH	P value	NAFLD	C-CH	P value
Gender (F/M)	13 / 11	11 / 18	ns	6 / 5	4 / 2	ns	9 / 9	8 / 16	ns
Age (yo)	58.5 ± 12.9	69.6 ± 9.2	<0.001	53.9 ± 14.2	73.8 ± 6.7	<0.001	58.8 ± 9.6	71.3 ± 8.0	<0.001
BMI (Kg/m^2)	28.6 ± 7.6	23.6 ± 2.8	<0.005	31.7 ± 9.9	25.3 ± 4.4	<0.05	28.9 ± 8.7	23.5 ± 2.9	<0.01
FBS (mg/dl)	118.0 ± 33.9	119.2 ± 46.7	ns	106.2 ± 32.1	87.0 ± 4.8	ns	112.0 ± 28.2	114.7 ± 44.5	ns
f-IRI (μU/ml)	18.4 ± 13.9	11.8 ± 7.5	ns	22.8 ± 17.9	10.5 ± 6.3	ns	19.2 ± 15.1	11.5 ± 7.6	ns
HOMA-IR	5.07 ± 3.80	3.50 ± 2.40	ns	5.55 ± 4.86	2.24 ± 1.31	ns	4.99 ± 4.10	3.35 ± 2.54	ns
HbA1c (%)	6.7 ± 1.8	6.5 ± 1.3	ns	6.0 ± 1.2	5.2 ± 0.5	ns	6.3 ± 1.3	6.6 ± 1.3	ns
1,5-AG (μg/ml)	13.4 ± 7.5	13.5 ± 9.3	ns	13.1 ± 5.4	16.9 ± 10.5	ns	14.9 ± 7.9	13.3 ± 8.4	ns
AST (IU/L)	69.3 ± 27.5	57.3 ± 27.1	ns	58.2 ± 26.5	60.7 ± 31.3	ns	64.7 ± 23.4	59.5 ± 28.3	ns
ALT (IU/L)	93.3 ± 52.0	43.9 ± 24.2	<0.001	62.7 ± 29.1	32.5 ± 11.3	<0.05	75.7 ± 36.0	46.3 ± 25.6	<0.005
ALP (IU/L)	285.1 ± 69.3	380.5 ± 164.1	<0.05	269.9 ± 72.3	362.2 ± 160.9	ns	285.3 ± 77.7	360.6 ± 160.8	ns
GGT (IU/L)	107.1 ± 80.9	65.8 ± 48.2	<0.05	104.1 ± 85.3	97.8 ± 159.4	ns	110.0 ± 86.5	72.8 ± 50.1	ns
T-Bil (mg/dl)	0.91 ± 0.41	1.11 ± 0.97	ns	1.15 ± 0.48	0.96 ± 0.43	ns	1.02 ± 0.48	0.88 ± 0.36	ns
TP (g/dl)	7.3 ± 0.5	7.0 ± 0.7	ns	7.2 ± 0.5	7.5 ± 1.0	ns	7.3 ± 0.5	7.0 ± 0.7	ns
ALB (g/dl)	4.3 ± 0.5	3.3 ± 0.7	<0.001	4.2 ± 0.4	3.6 ± 0.6	ns	4.2 ± 0.5	3.3 ± 0.8	<0.001
T-CHO (mg/dl)	196.8 ± 31.7	141.2 ± 29.6	<0.001	202.3 ± 39.0	156.0 ± 20.7	<0.05	192.6 ± 33.8	140.4 ± 26.0	<0.001
TG (mg/dl)	159.5 ± 61.5	98.1 ± 43.8	<0.001	128.8 ± 46.2	94.3 ± 22.4	ns	157.6 ± 65.8	103.6 ± 44.5	<0.05
RBC (X10^4 /μl)	457.1 ± 48.4	376.9 ± 51.6	<0.001	448.4 ± 52.2	350.8 ± 20.8	<0.001	453.6 ± 45.9	381.1 ± 46.1	<0.001
Hb (g/dl)	14.1 ± 1.3	12.2 ± 1.7	<0.001	14.1 ± 1.2	11.3 ± 0.8	<0.001	14.2 ± 1.3	12.4 ± 1.6	<0.001
PLT (X10^4/μl)	18.5 ± 7.0	11.8 ± 7.0	<0.005	15.5 ± 6.1	13.4 ± 5.4	ns	15.7 ± 6.4	12.8 ± 7.1	ns
WBC (X10^3/μl)	6.2 ± 1.4	4.8 ± 2.0	<0.01	5.4 ± 1.5	4.3 ± 1.3	ns	5.7 ± 1.4	5.0 ± 2.1	ns
Ⅳcollagen7S (ng/ml)	5.8 ± 2.1	9.6 ± 3.8	<0.001	5.9 ± 2.2	8.0 ± 2.0	ns	6.1 ± 2.1	9.1 ± 3.2	<0.005
P-3-P (U/ml)	0.8 ± 0.3	1.2 ± 0.3	<0.001	0.9 ± 0.3	1.2 ± 0.1	ns	0.8 ± 0.3	1.2 ± 0.3	<0.005
FIB-4 index	3.00 ± 2.14	7.52 ± 6.15	<0.005	3.53 ± 2.58	7.15 ± 5.44	ns	3.62 ± 2.29	6.69 ± 4.36	<0.05
PT (INR)	1.05 ± 0.09	1.17 ± 0.23	<0.05	1.09 ± 0.13	1.22 ± 0.22	ns	1.06 ± 0.10	1.14 ± 0.16	ns

Hyperglycaemia: maximum blood glucose of ≥180 mg/dl; Hypoglycaemia: minimum blood glucose of <70 mg/dl;
Excessive glycaemic variability: blood glucose swings of ≥110 mg/dl
